# Enhanced Cholinergic Activity Improves Cerebral Blood Flow during Orthostatic Stress

**DOI:** 10.3389/fneur.2017.00103

**Published:** 2017-03-20

**Authors:** Jorge M. Serrador, Roy Freeman

**Affiliations:** ^1^Department of Pharmacology, Physiology and Neuroscience, Rutgers Biomedical Health Sciences, Newark, NJ, USA; ^2^Department of Neurology, Beth Israel Deaconess Medical Center, Harvard Medical School, Boston, MA, USA; ^3^Cardiovascular Electronics, National University of Ireland Galway, Galway, Ireland

**Keywords:** cerebral blood flow, cholinergic, autonomic, orthostasis, orthostatic intolerance

## Abstract

Cerebral blood flow (CBF) and consequently orthostatic tolerance when upright depends on dilation of the cerebral vasculature in the face of reduced perfusion pressure associated with the hydrostatic gradient. However, it is still unclear if cholinergic activation plays a role in this dilation. To determine if enhancing central cholinergic activity with the centrally acting acetylcholinesterase inhibitor, physostigmine would increase CBF when upright compared to the peripherally acting acetylcholinesterase inhibitor, neostigmine, or saline. We performed a randomized double-blind dose-ranging study that took place over 3 days in a hospital-based research lab. Eight healthy controls (six women and two men, mean age, 26 years; range 21–33) were given infusions of physostigmine, neostigmine, or saline on three different days. Five-minute tilts were repeated at baseline (no infusion), Dose 1 (0.2 μg/kg/min physostigmine; 0.1 μg/kg/min neostigmine) and Dose 2 (0.6 μg/kg/min physostigmine or 0.3 μg/kg/min neostigmine), and placebo (0.9% NaCl). Cerebral blood velocity, beat-to-beat blood pressure, and end-tidal CO_2_ were continuously measured during tilts. Physostigmine (0.6 μg/kg/min) resulted in higher cerebral blood velocity during tilt (90.5 ± 1.5%) than the equivalent neostigmine (85.5 ± 2.6%) or saline (84.8 ± 1.7%) trials (*P* < 0.05). This increase occurred despite a greater postural hypocapnia, suggesting physostigmine had a direct vasodilatory effect on the cerebral vasculature. Cerebral hypoperfusion induced by repeated tilts was eliminated by infusion of physostigmine not neostigmine. In conclusion, this study provides the first evidence that enhancement of central, not peripheral, cholinergic activity attenuates the physiological decrease in CBF seen during upright tilt. These data support the need for further research to determine if enhancing central cholinergic activity may improve symptoms in patients with symptomatic orthostatic intolerance.

## Introduction

Orthostatic intolerance, which includes symptoms of lightheadedness, weakness, dizziness and presyncope, is a consequence of the inability to maintain cerebral perfusion when upright (1). While the peripheral vascular responses to orthostatic stress are well characterized, the cerebrovascular response is less clearly understood.

The upright posture results in a reduction in cerebral perfusion pressure due to the hydrostatic gradient (2), and thus cerebral resistance vessels must dilate to maintain cerebral blood flow (CBF). An inadequate dilatory response would impair flow. In fact, healthy individuals that develop orthostatic intolerance demonstrate greater decreases in CBF (3, 4), even when arterial pressure is maintained (4), suggesting cerebral vasoconstriction or inadequate cerebral vasodilation is present. The role of cerebral hypoperfusion in orthostatic intolerance patients of diverse causes remains unclear. Some studies have demonstrated greater decreases in CBF (3, 5) while others have not (6).

Cerebral vessels are densely innervated by parasympathetic nerves (7, 8) that could play a direct vasoregulatory role. Recently, it has been proposed that cholinergic activation could play an important role in control of the cerebral vasculature (9). Both animal (wide range of quadrupeds), primate and human studies have implicated parasympathetic nerves in cerebral vasodilation (7, 10, 11). Although several neurotransmitters are involved, there is evidence in humans that acetylcholinesterase inhibition leads to an increase in CBF without a change in metabolism while supine (12), supporting a direct vasodilatory role for cholinergic nerves. However, there are no reports on the role of the cholinergic system on CBF when upright.

To examine the role of the cholinergic system in cerebral vasodilation in healthy control participants during orthostatic stress, we administered the centrally acting, tertiary amine, cholinesterase inhibitor, physostigmine to enhance central cholinergic activity during orthostatic stress to determine if the CBF response to upright tilt would be improved. To control for the effects of increased peripheral cholinergic activity, we administered the quaternary amine, cholinesterase inhibitor, neostigmine, which does not cross the blood brain barrier. We hypothesized that cholinergic cerebral vasodilation would increase CBF in the upright posture.

## Materials and Methods

All procedures were approved by Beth Israel Deaconess Medical Center Committee on Clinical Investigations. All participants were consented by Dr. Jorge Serrador prior to participation in the study. Participants were given the consent form and the opportunity to read it. Then procedures were verbally explained by Dr. Serrador, and they were asked if they had any questions or concerns. After verbal confirmation was received that they understand the procedures and risks, they signed the consent form, and it was witnessed.

### Participants

Participants had no history of cardiopulmonary, renal, neurological or other systemic disease. Caffeine, alcohol, and heavy exercise were prohibited for 24 h prior to testing. A medical history, physical exam, and pregnancy test were performed on all participants.

### Drug Dosage

**Figure 1:**
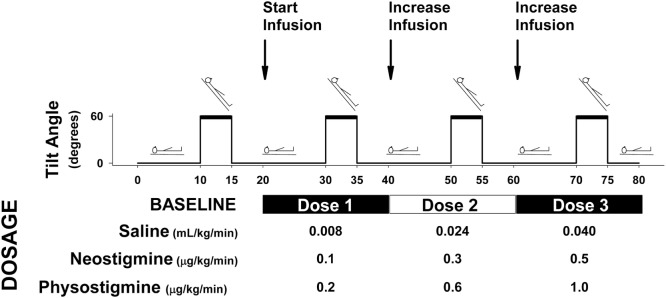
**Protocol diagram for physostigmine, neostigmine, saline infusion**. Protocol for each study session. Participants underwent four tilts consisting of 10 min supine, 5 min tilt, and 5 min recovery. Following each recovery period, infusion was started or increased (every 20 min). Participants were randomly assigned to drug condition, and infusions were performed in a double-blind fashion. Infusion doses are shown for each drug condition (neostigmine and physostigmine) and placebo control (saline).

Testing was double blind with participants returning on three separate occasions, with the drug received on each testing day randomly determined (saline, neostigmine, and physostigmine). Drugs were mixed at the research pharmacy and provided in an unlabeled bag so that study staff and participants were blind to what infusion was being performed. Each test day consisted of baseline and three dosage levels: baseline (no infusion), Dose 1 (0.2 μg·kg^−1^·min^−1^ physostigmine; 0.1 μg·kg^−1^·min^−1^ neostigmine), Dose 2 (0.6 μg·kg^−1^·min^−1^ physostigmine or 0.3 μg·kg^−1^·min^−1^ neostigmine), and Dose 3 (1.0 μg·kg^−1^·min^−1^ physostigmine or 0.5 μg·kg^−1^·min^−1^ neostigmine) or placebo (0.9% NaCl) for all three infusion levels (Figure 1).

### Cerebrovascular Reactivity

Cerebrovascular reactivity was assessed while breathing 8% CO_2_, 21% O_2_, balance nitrogen, and mildly hyperventilating for 2 min each. Two minute periods were chosen since previous work found the time constant for cerebral flow velocity responses to changes in end-tidal CO_2_ is ~45 s (13). Thus, the first 60 s allow for adaptation to the stimulus, and the second 60 s can assess steady state response. In addition, recent research has found that sustained hypercapnia or hypocapnia can result in changes in MCA diameter, but only after continual steady state periods longer than 2 min (14). Thus, using 2 min periods allows for assessing velocity changes without associated diameter changes.

### Tilt Testing

Each test session comprised four 5-min tilts (60°) preceded by 10 min supine rest and followed by 5 min of recovery. Following each recovery period, the infusion was increased so that participants received a total of three dose levels and four tilts (Figure 1). Participants remained upright for 5 min or until they developed presyncopal symptoms. Five-minute tilts with a 15 min supine recovery between tilts were used in order to complete all dosage levels within one session and to minimize the effect of repeated tilts on orthostatic tolerance. A prior report suggests that six repeated 10 min tilts with 30 s supine in between results in reduced orthostatic tolerance (15). Heart rate (HR) (Datex Cardiocap II), beat-to-beat blood pressure (BP) (Finometer), cerebral flow velocity in the middle cerebral (CFV_MCA_), and anterior cerebral (CFV_ACA_) arteries (transcranial Doppler, Multi-Dop X4), and end-tidal CO_2_ (Datex-Ohmeda) was continuously recorded.

#### Data Analysis

Signals were sampled at 500 Hz and processed using data acquisition (WinDaq) and analysis (MATLAB) software. Cerebrovascular reactivity was determined by plotting cerebral flow velocity vs end-tidal CO_2_ during testing including a 6 s delay between end-tidal and flow velocity changes. Plots were created of breath-by-breath end-tidal CO_2_ vs CFV was created over both the baseline, hypercapnia and hypocapnia periods. Cerebrovascular reactivity was determined as the linear best fit for the data.

BP_brain_ (BP at heart level minus hydrostatic gradient) was used to reflect cerebral perfusion pressure since intracranial pressure remains relatively unchanged (2). CFV was normalized to baseline (supine rest prior to first tilt) to better reflect changes in CBF (16). Estimated cerebrovascular resistance (CVR) was derived by dividing BP_brain_ by CFV. To minimize probe placement effects, TCD operators and probe depths were kept constant.

### Statistics

To examine the role of cholinergic activity on the cerebrovascular response to tilt, comparisons were made between drugs as well as dose and body position, i.e., supine vs upright. A three-way repeated-measures ANOVA was performed (Drug × Dose × Tilt), with a Bonferroni *post hoc* analysis. Mean values during last 3 min of tilt were compared to baseline (mean of last 5 min supine prior to tilt). Data are presented as mean ± SEM, *p* < 0.05 considered significant.

## Results

### Participant Characteristics

Eight participants (6 women and 2 men, mean age, 26 years; range 21–33 years, 67.7 ± 15.2 kg, 166.7 ± 4.7 cm, mean ± SD) completed 12 tilts over 3 visits without developing presyncope.

### Effect of Repeated Orthostatic Stress during Saline Infusion

**Figure 2:**
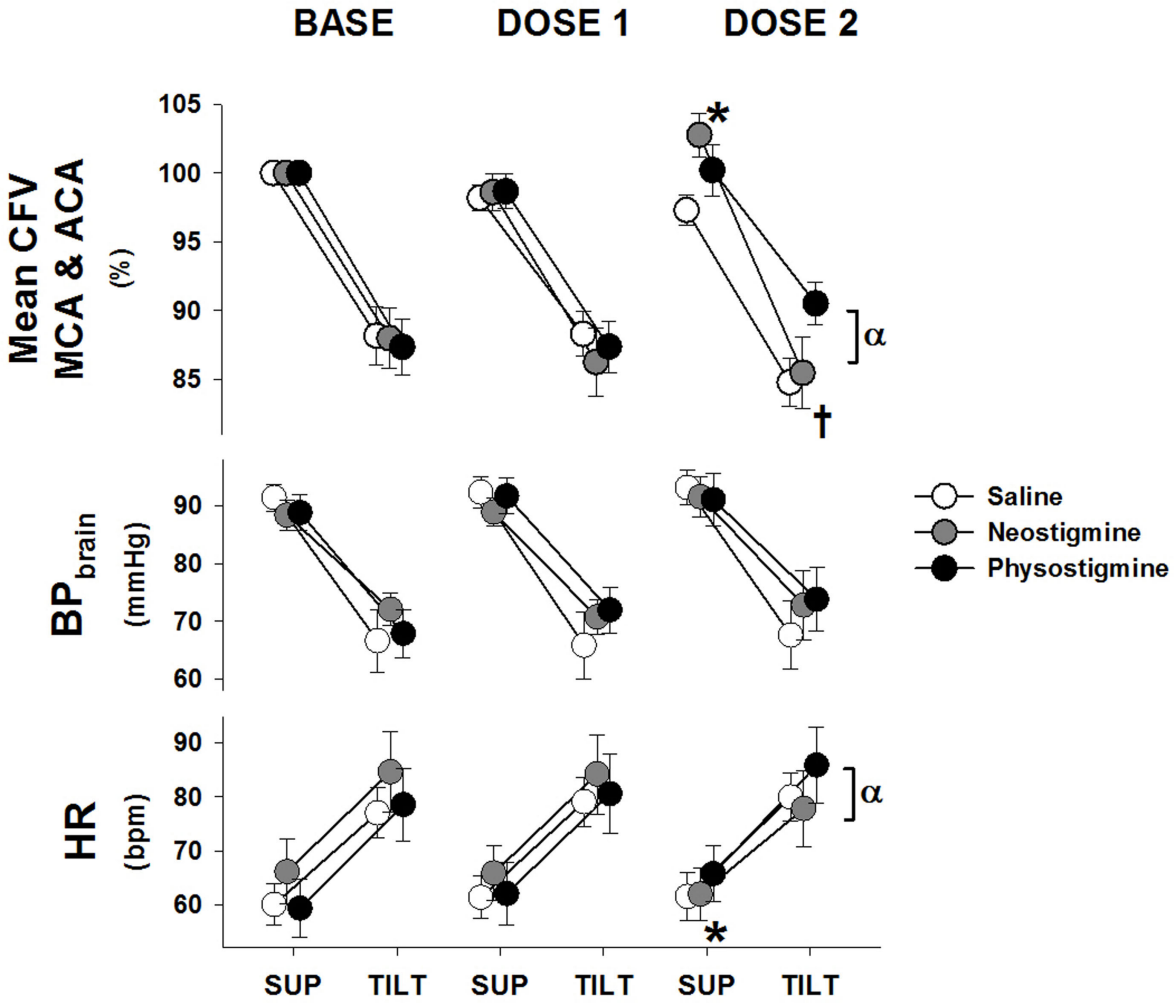
**Cardiovascular response to tilt during physostigmine, neostigmine, and saline infusion**. Changes in mean cerebral flow velocity for both arteries (CFV), blood pressure corrected to brain level (BP_brain_), and heart rate (HR) from supine (mean of 6–10 min) to 60° head up tilt during infusion of saline (white), neostigmine (gray), or physostigmine (black) at Baseline (no infusion), Dose 1, and Dose 2. *A significant difference from baseline supine values (*P* < 0.05). ^†^Significantly lower CFV while upright compared to Tilt 1 for saline and neostigmine only (*P* < 0.05). ^α^Significant effect of drug at that dose (*P* < 0.05). All tilt values were significantly different from supine (*P* < 0.005).

**Figure 3:**
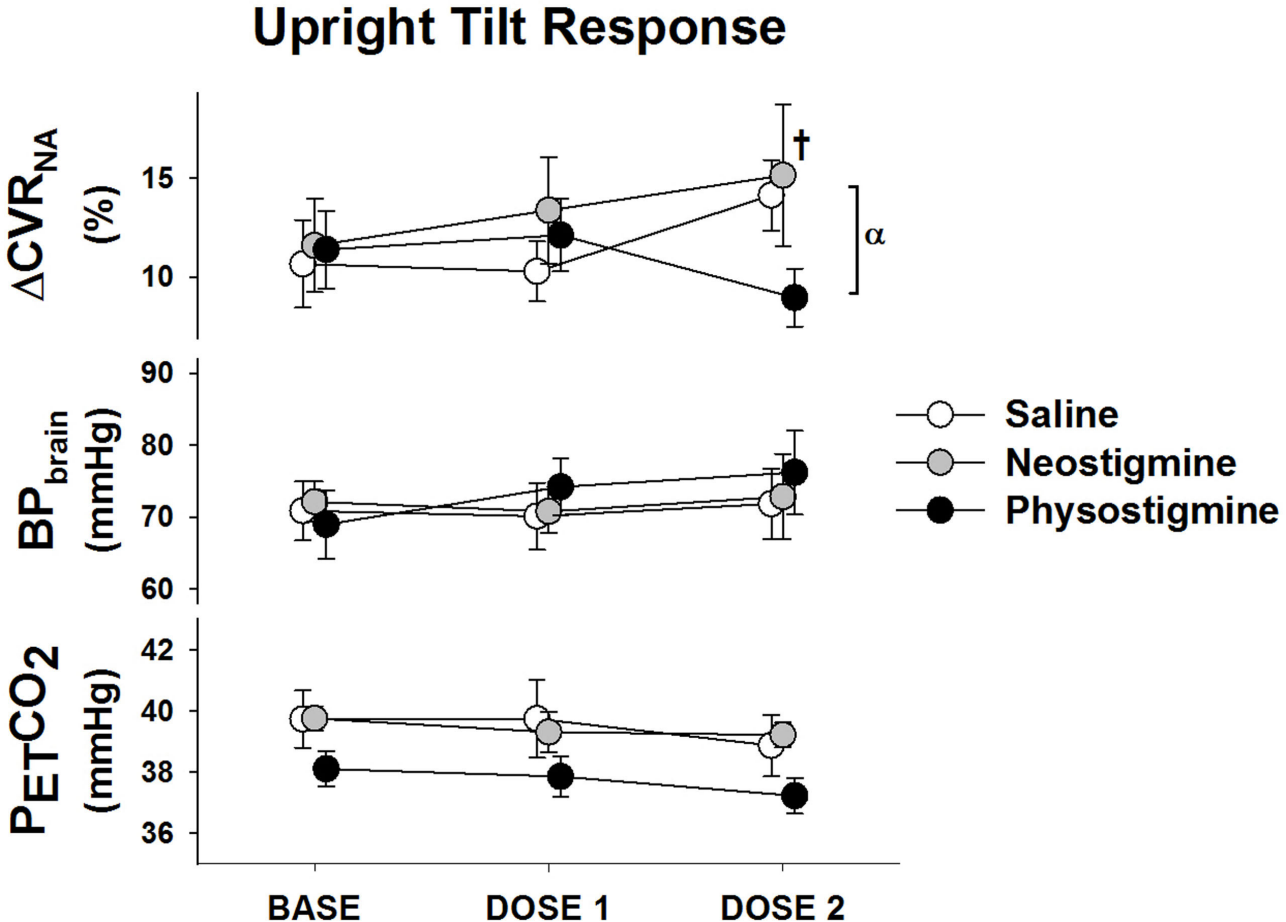
**Non-autoregulatory response to infusion**. The non-autoregulatory cerebrovascular resistance (CVR_NA_), blood pressure corrected to brain level (BP_brain_), and end-tidal CO_2_ (P_ET_CO_2_) response to 5 min of 60° head up tilt during infusion of saline (white), neostigmine (gray), or physostigmine (black) at Baseline, Dose 1, and Dose 2. ^†^Significantly higher CVR_NA_ while upright compared to Tilt 1 for saline and neostigmine only (*P* < 0.05). ^α^Indicates significant effect of drug at that dose (*P* < 0.05).

**Figure 4:**
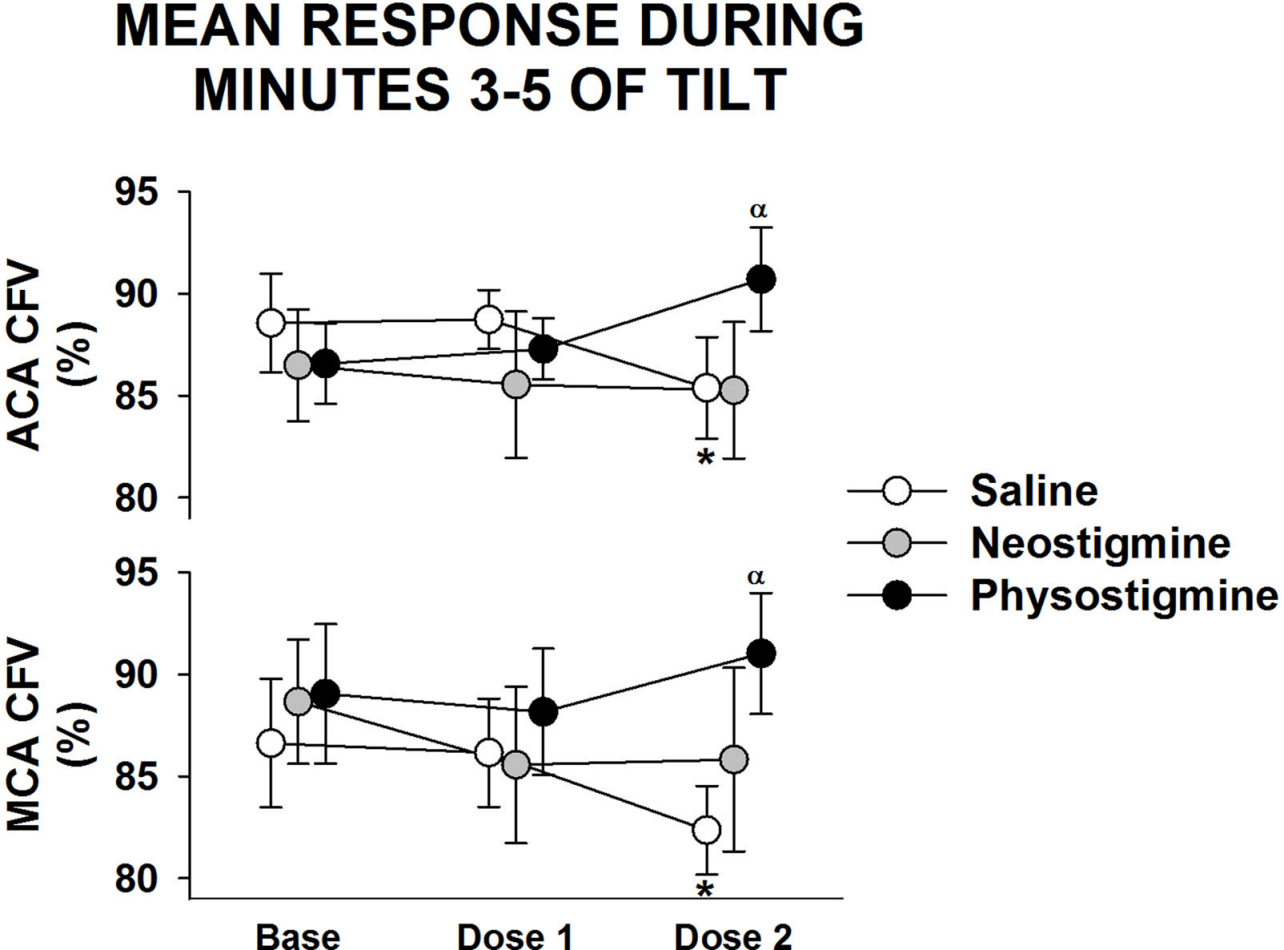
**Regional cerebral blood flow response to infusion**. Anterior cerebral artery (ACA) and middle cerebral artery (MCA) cerebral flow velocity (CFV) response during 5 min of 60° head up tilt during saline infusion and during Dose 1 and Dose 2 infusion of either neostigmine or physostigmine. There were no significant differences between arteries in responses to either dosage or drug. *A significant difference from baseline tilt values (*P* < 0.05). ^α^Significant effect of drug at that dose (*P* < 0.05).

Blood pressure while upright was similar during all tilts as was HR (Figures 2 and 3, open circles). Repeated orthostatic stress resulted in significantly reduced cerebral flow velocity while upright during Tilt 3 (Figure 4). It also resulted in slightly reduced end-tidal CO_2_ during the third tilt compared to the first (<1 mmHg difference), see Table 1.

**Table 1:** **Response of blood pressure (BP), cerebrovascular resistance (CVR), and end-tidal CO_2_ to repeated tilts during saline, neostigmine, and physostigmine infusion**.

			No infusion (Tilt 1)			Dose 1 (Tilt 2)			Dose 2 (Tilt 3)		
			Baseline	Tilt	Recovery	Baseline	Tilt	Recovery	Baseline	Tilt	Recovery
Saline	BP (mmHg)	Systolic	132.2 ± 3.8	128.8 ± 4.7	132.2 ± 3.9	133.9 ± 4.8	127.8 ± 5.9	134.5 ± 4.8	134.8 ± 4.6	130.2 ± 5.7	134.9 ± 3.8
		Diastolic	71.1 ± 1.9	72.0 ± 3.6	69.7 ± 1.8*	71.7 ± 1.8	71.2 ± 3.7	70.5 ± 1.9*	72.4 ± 2.3	72.7 ± 3.8	71.4 ± 1.9*
	CVR (mmHg/%)		0.93 ± 0.02	0.82 ± 0.03*	0.96 ± 0.01	0.96 ± 0.02	0.78 ± 0.02*	0.98 ± 0.02	0.98 ± 0.02	0.84 ± 0.03*	1.00 ± 0.02
	End-tidal CO_2_ (mmHg)		41.0 ± 0.9	39.8 ± 0.9*	41.1 ± 1.0	41.1 ± 0.9	39.7 ± 1.0*	40.9 ± 0.9	40.9 ± 0.7	38.9 ± 0.9*^,^^†^	41.1 ± 1.1
Neostigmine	BP (mmHg)	Systolic	128.7 ± 3.4	134.0 ± 2.5	126.2 ± 3.8	128.6 ± 3.0	131.5 ± 2.7	126.8 ± 1.8	130.0 ± 3.3	134.1 ± 4.1	129.6 ± 4.0
		Diastolic	68.3 ± 2.7	79.9 ± 2.1*	67.2 ± 2.6	69.1 ± 2.5	79.2 ± 1.3*	68.0 ± 2.3	72.4 ± 4.2	81.0 ± 4.5*^,^^‡^	69.7 ± 3.3
	CVR (mmHg/%)		0.89 ± 0.02	0.75 ± 0.01*	0.90 ± 0.02	0.90 ± 0.02	0.72 ± 0.02*	0.90 ± 0.02	0.89 ± 0.03	0.74 ± 0.02*	0.92 ± 0.02
	End-tidal CO_2_ (mmHg)		42.3 ± 0.8	39.9 ± 0.3*	42.0 ± 0.9	42.3 ± 0.7	39.4 ± 0.6*	42.2 ± 0.8	42.0 ± 0.7	39.5 ± 0.4*^,^^†^	41.7 ± 0.6
Physostigmine	BP (mmHg)	Systolic	132.2 ± 3.9	130.7 ± 4.5	130.9 ± 4.3	132.8 ± 4.1	133.0 ± 5.2	131.3 ± 4.6	132.4 ± 5.6	134.9 ± 7.0	131.8 ± 6.0
		Diastolic	67.2 ± 3.0	73.8 ± 4.5*	67.6 ± 2.7	71.2 ± 2.9	78.7 ± 3.7*	70.3 ± 3.4	70.4 ± 4.2	80.6 ± 5.0*	72.4 ± 3.8
	CVR (mmHg/%)		0.90 ± 0.03	0.75 ± 0.02*	0.92 ± 0.03	0.94 ± 0.03	0.73 ± 0.03*	0.96 ± 0.04	0.92 ± 0.05	0.77 ± 0.02*	0.90 ± 0.05
	End-tidal CO_2_ (mmHg)		42.0 ± 0.6	38.0 ± 0.6*	41.5 ± 0.8	41.4 ± 0.5	37.8 ± 0.7*	40.9 ± 0.5	40.4 ± 0.5	37.1 ± 0.6*^,^^†^^,^^‡^	39.5 ± 0.9^‡^

*Values represent 5 min means during supine baseline (Baseline), last 3 min of 60° upright tilt (Tilt), and supine recovery (Recovery)*.

*Significant difference from Baseline (*P* < 0.05).

*^†^Significant difference from Tilt 1 (*P* < 0.05)*.

*^‡^Significant from saline condition (*P* < 0.05)*.

### Effect of Acetylcholinesterase Inhibitors on the Orthostatic Response

#### Neostigmine

Supine BP and end-tidal CO_2_ were unaffected by dosage level during the 5 min prior to tilt (Table 1). In contrast, CFV in both arteries demonstrated a small but significant increase at Dose 2 while HR decreased during the same supine period. During tilt at Dose 2, neostigmine resulted in lower cerebral flow velocity as well as lower HRs (Figure 2) and slightly lower end-tidal CO_2_ (<1 mmHg, Table 1). Lower cerebral blood flow velocity and end-tidal CO_2_ were also seen during the third tilt with saline infusion, and thus these changes may be have been due to repeated tilts rather than neostigmine. Mean arterial pressure (MAP), when upright, was unaffected by dosage but diastolic BP increased. During Dose 3, the trial was stopped in one participant due to the development of nausea.

#### Physostigmine

Supine BP, end-tidal CO_2_ and cerebral flow velocity were unaffected by dose level during the 5 min prior to tilt (Figure 2 and Table 1). In contrast, CFV and HR increased at Dose 2. During tilt at Dose 2, cerebral flow velocity values were higher compared to baseline tilt (Figure 2). This increased CBF velocity occurred despite there being no difference between baseline and Dose 2 tilts in BP or HR. Similar to saline and neostigmine, end-tidal CO_2_ was slightly lower during Dose 2 compared to baseline tilt (<1 mmHg, Table 1). The increase in CFV was present across arterial territories (i.e., both middle and anterior cerebral arteries) and during supine recovery (Figure 4).

Cerebrovascular resistance decreased during tilt in all conditions (Table 1) but not sufficiently to maintain flow at baseline levels. Since CVR changes are due to both autoregulatory responses to pressure changes and neurogenic vasoregulation, we examined the non-autoregulatory component of the CVR response (CVR_NA_). This was determined by subtracting the autoregulatory CVR (CVR necessary to maintain 100% of baseline flow during pressure changes) from the actual CVR obtained during each tilt. Since measures of autoregulation (transfer function gains) demonstrated no changes with infusion (data not shown), we assumed autoregulation was intact. Comparison of the upright response during the first three tilts demonstrated that in contrast to the saline and neostigmine Dose 2 tilts where CVR_NA_ was greater, during Dose 2 physostigmine CVR_NA_ was lower, suggesting improved vasodilation, which would explain the better maintenance of cerebral flow velocity (Figure 3). In addition this would suggest that the physostigmine was able to counteract the detrimental effect of repeated tilts that resulted in reduced flow velocity and increased resistance during Tilt 3 in the saline and neostigmine groups. Dose 3 of the physostigmine trial was terminated in seven of eight participants due to the development of nausea and thus was not included in the analysis.

### Response of Regional CBF

**Figure 5:**
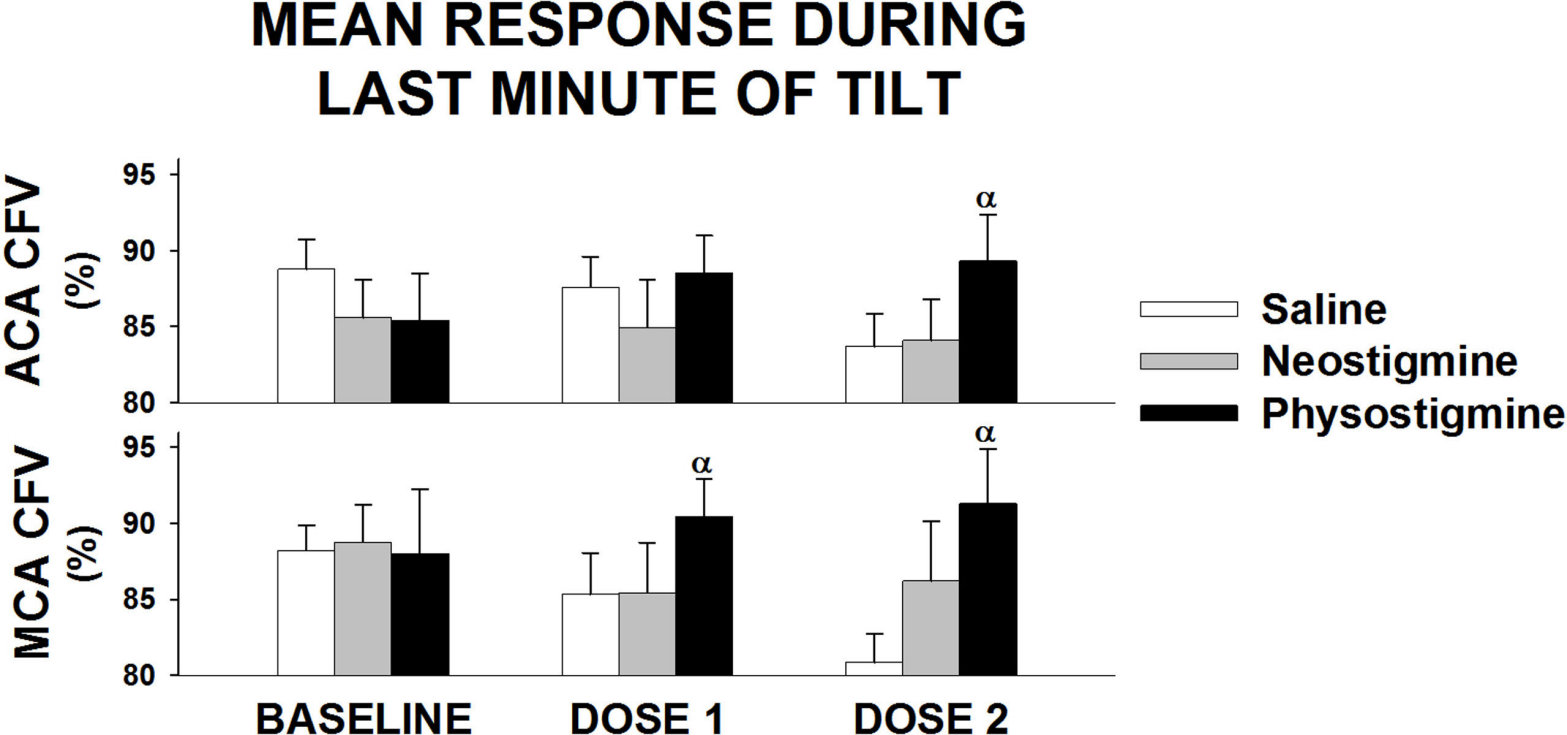
**Regional cerebral blood flow response to infusion during last minute of tilt**. Anterior cerebral artery (ACA) and middle cerebral artery (MCA) cerebral flow velocity (CFV) response during last minute of 5 min of 60° head up tilt during saline infusion and during Dose 1 and Dose 2 infusion of either neostigmine or physostigmine. There were no significant differences between arteries in responses to either dosage or drug. ^α^Significant effect of drug at that dose (*P* < 0.05).

Examination of middle cerebral and anterior cerebral artery velocity demonstrated that both arteries had similar changes associated with repeated tilt for both neostigmine and physostigmine (Figure 4). Decreases in middle cerebral artery velocity tended to be greater than those seen in the anterior cerebral artery during both repeated saline and neostigmine tilts, although this trend did not reach significance. Interestingly, middle cerebral artery velocity was improved during both Dose 1 and Dose 2 of physostigmine in the last minute of tilt (Figure 5). Probe placement was consistent across days; no difference in cerebral flow velocity during first baseline, regardless of trial (*P* = 0.80). Cerebrovascular reactivity was not significantly different between trials (*P* = 0.50) and did not differ between arteries (*P* = 0.70).

## Discussion

This study provides the first evidence that enhancement of central, not peripheral, cholinergic activity attenuates the physiological decrease in CBF seen during upright tilt. We also found that enhancing central cholinergic activity (*via* physostigmine) attenuates the decrease in CBF associated with repeated exposure to short duration orthostatic stress.

Assumption of the upright posture is associated with a number of adaptations to maintain arterial pressure in response to translocation of blood in the dependent circulation. Parasympathetic activity is attenuated, and sympathetic outflow increases, leading to increased HR and peripheral vasoconstriction to maintain arterial pressure. In addition, the cerebral vasculature is now above the heart, and perfusion pressure is reduced ~20 mmHg (2). Thus, to maintain CBF in the upright position, cerebral vessels must dilate. If cerebral vessels are unable to dilate and cerebral hypoperfusion occurs, syncope will result.

Several *animal* models provide evidence that parasympathetic pathways are implicated in cerebral vasodilation (7, 10). Studies document that parasympathetic activation causes increased CBF independent of metabolism suggesting a direct vasodilatory role (10, 17). Similarly removal of the pterygopalatine ganglion interrupts parasympathetic pathways and causes reductions in CBF in rats (18). In addition, enhancement of cholinergic activity using physostigmine improves the functional increase in CBF in the somatosensory cortex during vibrotactile stimulation [determined by positron emission tomography (PET)] in elderly monkeys (19). Similarly, in young-adult monkeys, impairing cholinergic activity using scopolamine abolishes the functional increase in CBF during vibrotactile stimulation and enhancing cholinergic activity *via* physostigmine reinstates it. In *humans*, several lines of evidence suggest a role for cholinergic inputs. For example, regional CBF, as measured by PET or SPECT, increases during stimulation of the vagus nerve (11, 20) or inhibition of acetylcholinesterase with physostigmine (12, 21–24). While flow increased with physostigmine in these studies, metabolism was unchanged (12), further supporting a direct vasodilatory effect. There is also evidence that administration of scopolamine causes a decrease in CBF that is reversed by physostigmine but not neostigmine, supporting a role for central cholinergic regulation of CBF (25). Neostigmine, which does not cross the blood brain barrier, also did not improve the CBF response in our studies. While some work has found no change in CBF with physostigmine administration (21), or decreases in global CBF in healthy controls (26), the later study also reported a tendency for global CBF to increase in veterans with Gulf War Illness and cognitive dysfunction. Veterans with Gulf War Illness have also been found to have cholinergic autonomic dysfunction (27). Thus, the increase in CBF with physostigmine infusion while supine in these veterans may have been the result of the physostigmine enhancing cholinergic activity from impaired to normal levels. Further work in this group would be necessary to confirm this.

However, one important differentiation between the current work and all of the previous work mentioned was that none of the previous studies have examined the upright response. Without this orthostatic stress, the improvement in CBF with cholinergic enhancement would have been missed.

Consistent with previous work (15), we found that repeated tilts (in both the saline and neostigmine conditions) caused a greater reduction in CBF when upright and increased CVR (see Tilt 3, Figures 2 and 3). In contrast, during physostigmine infusion, there was increased CBF compared to previous tilts. Thus, the increase in CBF during Tilt 3 with physostigmine infusion occurred despite the parallel detrimental effect of repeated tilts on cerebral perfusion. Furthermore, physostigmine also attenuated the greater cerebral hypoperfusion during supine recovery (Table 1). However, the mechanism for this detrimental effect of repeated orthostatic stress remains unknown.

While our data demonstrate that pharmacological enhancement of central cholinergic activity improves CBF during orthostasis, it is not known whether there is physiological activation of the central cholinergic system during orthostatic stress; particularly since peripheral cholinergic activity decreases during orthostatic stress. Several lines of evidence support differential autonomic outflow to individual vascular beds, for example, sympathetic activation causes mesenteric vasoconstriction with hindquarter vasodilation in rats (28) and femoral vasoconstriction without affecting CVR in humans (29). These data lend some support to the possibility that there is a physiological increase in cerebral cholinergic activity in response to orthostatic stress despite reduced peripheral parasympathetic activity.

The mechanisms that underlie neurogenic cerebral vasodilation are not fully resolved. Preclinical and clinical evidence support a role for the cholinergic system; (1) cerebral vessels have dense cholinergic innervation (7); (2) stimulation of brainstem parasympathetic nuclei, cholinergic nerves, and basal forebrain cholinergic nuclei leads to cerebral vasodilation that is potentiated by physostigmine and attenuated by atropine (30); and (3) central acetylcholinesterase inhibition with physostigmine enhances CBF (10). However, several prior studies suggest that nitric oxide is the primary cerebral vasodilator and that acetylcholine plays a modulating or facilitatory role (31, 32).

While no previous work has examined the effect of physostigmine on the cerebrovascular response to orthostatic change, pyridostigmine, which does not cross the blood brain barrier, improves standing BP in patients with orthostatic hypotension due to autonomic failure (33) and attenuates the HR increase in patients with postural tachycardia syndrome (34). It is unclear whether pyridostigmine affected CVR in these patients since CBF was not measured. In contrast, we found no significant change in BP or improvement in CBF during tilt in healthy controls during peripheral cholinergic activity enhancement with neostigmine.

Thus, the role played by cerebral cholinergic vasodilatory mechanisms in the features of orthostatic tolerance in autonomic failure patients is not known. The present data raise the possibility that cholinergic activation may enhance CBF. Whether this enhancement of CBF could then improve symptoms of orthostatic intolerance in this patient population needs to be examined. Further work is required to confirm this in patients and determine the dose–response given the narrow therapeutic window.

However, some support for a role for CBF in orthostatic intolerance in these patients is provided by examining current treatments. Midodrine, which has been found to be effective for treating orthostatic intolerance, has also been shown to increase CBF in patients with chronic hypotension (35) and spinal cord injured patients (36). Similarly, dihydroxyphenylserine has also been used to treat orthostatic intolerance and has also been found to increase regional CBF (37). Other treatments, such as pyridostigmine, a peripheral acetylcholinesterase inhibitor that does not cross the blood brain barrier, have not been found to affect CBF in animals (38). All of these treatments have also been found to increase BP, which may also underlie the increase in CBF. Thus, further work in orthostatic intolerance patients examining many variables including BP, cardiac output, and CBF would be necessary to better understand the role of CBF in orthostatic intolerance.

It is interesting that Does 2 of physostigmine resulted in higher HRs when upright. We would expect that physostigmine should reduce HR since it enhances cholinergic inputs to HR control. However, previous work in humans has also found that physostigmine infusion results in increased HRs (39, 40). The presumed mechanism is central activation of the hypothalamic–pituitary–adrenocortical axis as well as increased sympathetic outflow to the heart.

Several other explanations for the increase in CBF can be considered. If participants had impaired autoregulation, an increase in MAP during physostigmine infusion could have resulted in increased cerebral flow; however, there was no significant increase in pressure seen. Also, we did not find any indications of impaired autoregulation, which is consistent with evidence in primates where direct parasympathetic stimulation results in cerebral vasodilation without affecting autoregulation (17).

In addition, middle cerebral artery flow was increased during the last minute of tilt during the lowest physostigmine dose (Figure 5), suggesting that lower doses of physostigmine could be effective during longer periods of orthostatic stress. Further, work during longer tilts and testing the effects of lower doses of physostigmine on orthostatic tolerance is needed.

One limitation of this study is we were unable to assess sex differences due to the small number of participants (six women vs two men). Previous research has demonstrated CBF regulation may differ between women and men with older women demonstrating better cerebral autoregulation (41) and young women showing greater CBF (42, 43). In addition, CBF appears to be affected by time of cycle (44, 45). Since we did not control for time of cycle, we cannot examine possible cycle effects on the response. Further, work is needed to determine if sex differences and time of cycle affect the response to physostigmine.

### Summary

This work provides the first evidence that increasing central cholinergic activity improves the maintenance of CBF in the upright posture in healthy participants. These data raise the interesting possibility that use of centrally acting acetylcholinesterase inhibitors may improve symptoms in patients with symptomatic orthostatic intolerance. Further, work in patient populations is necessary to determine whether enhancing central cholinergic activity will improve CBF and if this could possibly reduce symptoms of orthostatic intolerance.

## Author Contributions

JS and RF conceived and designed the study, interpreted the data, and wrote the manuscript. JS performed the study, analyzed the data, had full access to all of the data in the study, and took responsibility for the integrity of the data and the accuracy of the data analysis.

## Conflict of Interest Statement

The authors declare that the research was conducted in the absence of any commercial or financial relationships that could be construed as a potential conflict of interest.
